# An Ethical Analysis of Pharmacy Benefit Manager (PBM) Practices

**DOI:** 10.3390/pharmacy7020065

**Published:** 2019-06-14

**Authors:** Jacob J. Drettwan, Andrea L. Kjos

**Affiliations:** College of Pharmacy and Health Sciences, Drake University, Des Moines, IA 50311, USA; jacob.drettwan@drake.edu

**Keywords:** pharmacy benefit manager (PBM), ethics, pharmaceutical regulation, health care policy, decision-making, organizations, ethical models, code of ethics, pharmacist

## Abstract

The high costs associated with pharmaceuticals and the accompanying stakeholders are being closely evaluated in the search for solutions. As a major stakeholder in the U.S. pharmaceutical market, the practices of pharmacy benefit manager (PBM) organizations have been under increased scrutiny. Examples of controversial practices have included incentives driving formulary status and prohibiting pharmacists from disclosing information on lower-cost prescription alternatives. Ethical investigations have been largely omitted within the debate on the responsibilities of these organizations in the health care system. Ethical analysis of organizational practices is justified based on the potential impact during health care delivery. The objective of this study was to analyze several specific PBM practices using multiple ethical decision-making models to determine their ethical nature. This study systematically applied multiple ethical decision-making models and codes of ethics to a variety of practices associated with PBM-related dilemmas encountered in the pharmaceutical environment. The assessed scenarios resulted in mixed outcomes. PBM practices were both ethical and unethical depending on the applied ethical model. Despite variation across applied models, some practices were predominately ethical or unethical. The point of sale rebates were consistently determined as ethical, whereas market consolidation, gag clauses, and fluctuation of pharmacy reimbursements were all predominantly determined as unethical. The application of using provider codes of ethics created additional comparison and also contained mixed findings. This study provided a unique assessment of PBM practices and provides context from a variety of ethical perspectives. To the knowledge of the authors, these perspectives have not been previously applied to PBM practices in the literature. The application of ethical decision-making models offers a unique context to current health care dilemmas. It is important to analyze health care dilemmas using ethics-based frameworks to contribute solutions addressing complexities and values of all stakeholders in the health care environment.

## 1. Introduction

The costs of pharmaceuticals as a portion of health care spending has continued to vex the United States (U.S.) as well as the rest of the world for several decades [1]. This issue has prompted vigorous discussion from a variety of stakeholders in the attempt to find agreeable solutions to a seemingly insolvable problem [2]. Discussions with experts and stakeholders have included government payers, health insurance organizations and pharmaceutical manufacturers. The most common frameworks considered in solutions are economic and legal rationales [3]. Economic defense of high costs often point towards the capitalistic free market and hence, theory of supply and demand. Further, legal considerations rely on explanations related to intellectual property rights, patents, proprietary information, obligations to shareholders, as well as complexities of government policies, regulation and oversight [1,2]. As an example of legal complexities, the United States health care practices (including health insurance law) are regulated at the state level (e.g., New York will differ from California), in contrast to uniformity at a national level [4]. This results in a variety of co-existing regulations depending on the regional location of the organization, the care provider, or the patient. Even beyond the U.S., legally sanctioned intellectual property rights and patent laws for pharmaceuticals are not always consistent across countries, resulting in complexities in the world-wide market [5].

In the U.S., the fiduciary issue of high spending on pharmaceuticals is one that involves specific interrelated stakeholder organizations including manufacturers, wholesalers, pharmacies, pharmacy benefit managers (PBMs), and plan sponsors (i.e., health insurance organizations) [6,7]. Briefly, these organizations and their relationships include the following: Pharmaceuticals are developed and made by manufacturers, delivered to pharmacies by wholesale distributors, obtained by patients at pharmacies where there is often reduced pricing according to coverage by plan sponsors [6,7]. PBMs are organizations that developed to provide added value and reduced costs by specifically managing the pharmaceutical benefit on behalf of a plan sponsor. In addition to using formularies and utilization requirements to control cost and incentivize cost-effective medication use, PBMs also negotiate lower prices from manufacturers [8]. This practice is commonly known as rebating. Although ideally a cost-saving tool, rebate levels are confidential and the actual cost savings is unknown. Under normal market conditions this would not be problematic, however, there is evidence to suggest that the most cost-effective pharmaceuticals are not being incentivized due to how PBMs sometimes place higher-priced pharmaceuticals as the preferred status on formularies and patients may spend more than they would for lower cost, equally effective alternatives [8]. Recently, PBM business practices such as rebating have begun to receive public scrutiny as the U.S. looks for solutions to the problem of high costs of pharmaceuticals [9,10,11,12,13]. Additional business practices undergoing scrutiny include exclusion lists, gag clauses, rapid fluctuation in pharmacy reimbursement rates, market consolidation and point of sale rebates [14]. In brief, exclusion lists are a way to specify non-coverage of certain medications, that is, it is a list of prescription medications that are excluded from any plan sponsor coverage. It has been noted that these exclusion lists are growing longer, in particular, for new or more expensive prescription medications [15]. A lesser-known policy referred to as gag clauses are in reference to a commercial contract between a PBM and pharmacies prohibiting a pharmacist’s ability to inform a patient whether or not a prescription has alternative purchasing options [16]. Another concern with PBMs has been the fluctuation of reimbursements through the use of redirect and indirect remuneration (DIR) fees assessed to pharmacies, retrospectively, dependent upon pharmacy performance or upon changes in market prices [17]. The practice known as point of sale rebates refers to PBM contracts with a discount program to offer cash-paying consumers an ability to purchase prescription medications at prices lower than a pharmacy’s list price of the medication [18] and circumvents the effectiveness of utilization incentives towards lower cost alternatives. Finally, as in many industries, the market consolidation of PBMs with other types of organization in the form of vertical integration challenge the intent of legal anti-trust statutes [7] (pp. 30–32), further attracting public scrutiny.

Health providers often find themselves as reluctant mediators between patients and high cost pharmaceuticals. Generally, provider involvement includes prescribing, educating, dispensing, and even administration of pharmaceuticals pursuant to laws regulating the scope of practice for various types of health professions. Specific to physicians, it has been argued that “physicians often neglect, or are simply unaware of, their role as economic agents for patients. Physicians play a crucial role in controlling prices and enabling patients to access affordable therapies” [19] (p. 9). In contrast, many community pharmacists are well versed in advocating for patient access to medications in the commercial environment of high costs [20].

It is pertinent that the experts and researchers continue work to find agreeable solutions to the problem of high costs of pharmaceuticals. To frame these discussions, an ethical framework would stand in contrast to the use of an economic or legal framework. Although there are a number of ethical theories worthy of consideration, in comparison to other frameworks, the ethical dialog uses concepts such as avoiding harm, duty to patients, virtues, justice, or the common good. Interestingly, stakeholder organizations and their corresponding practices described above, have remained largely uncriticized in the ethical literature, with the only exception being pharmaceutical manufacturers [19,21]. However, PBMs have been showing signs of increased scrutiny by private nonprofit health foundations [8], the U.S. government [7,14], professional pharmacy organizations [20,22] and in the economic literature [23,24]. The increased scrutiny directed toward PBMs has resulted from shielded business practices that may raise ethical concerns. For example, the literature has debated whether the reduced prices for some result in increased prices for others [13,19]. More specifically, PBMs may be causing a form of price-shifting between stakeholders, resulting in increased profits for manufacturers and PBMs, reduced revenues for pharmacies, reduced prices for some patients and plan sponsors, and increased prices for other patients and plan sponsors [23].

An ethical-oriented framework is one that is easily relatable to the case-based decisions of health care providers (e.g., pharmacists or physicians) [25]. Ethical principles are used in health care to provide a moral grounding for the day to day work of providers. The study of ethics remains an important part of education and culture for nearly all care providers charged with supporting the health of individuals in society [26,27]. Codes of ethics exist for clinical health professionals, and are intended to guide decision-making in the face of competing interests, social dilemmas, or conflicts encountered during the provision of care [28]. Although the study and application of ethics remain at the core of health professionals’ conduct, some have begun to question the degree to which the application of ethical principles may be in conflict with daily work requirements within the health care system, especially when balancing fiscal considerations [29]. Some base a growing concern for physician burnout and failing mental health on a kind of moral crisis [30]. Further, an application of a concept previously used for combat veterans known as moral injury has been used in the context of health care and posited as resulting from providers feeling as if their work is dissonant with their own moral and ethical principles [31].

Although there is an abundance of literature in the application of ethics in health care related to the provider-patient interactions, there has been substantially less attention on how health care organizations impact providers during the delivery of care [32]. In fact, organizational ethics is considered as an adjacent field to more typical bioethics applications in health care. However, some ethical theory work has shown new approaches using contextual differences to shape discussion when a dilemma includes both clinical and organizationally ethical standpoints [33]. As the dilemma of the high costs of pharmaceuticals is situated most prominently at an organizational level, it has been rarely considered through the more common bioethical (i.e., provider-patient focused) lens in the health care literature. Arguments to address this gap have used organizational ethics to demonstrate that stakeholder organizations can make decisions from a moral point of view in cooperation with the broader health care environment [21]. In addition, some have discussed a need for a cross-over between ethics and other fields, such as economics, to avoid economic-based solutions that inadvertently compound ethical concerns [19].

Specific to the field of pharmacy, a comprehensive review of the theory of pharmacy ethics found the existence of very little formal literature on philosophical values or frameworks specific to pharmacy [34]. Rather, this review found application of traditional medically-based bioethical concepts to pharmacy-oriented practice scenarios, as well as the application of professional codes to direct the behavior of pharmacists. Therefore, research on pharmacy ethics presents an area ripe for investigation due to the constant balance of the clinical aspects of patient care with commercial aspects of practice, especially in the community pharmacy settings [34]. Some limited theory-based research in this area found health care organizations do shape the ethical decision-making of pharmacists [35]. For example, some research has found that professional hierarchies, closeness (or distance) from patient care, or daily gatekeeping (i.e., roles balancing clinical care with fiscal responsibility) can require a great deal of provider moral contemplation [36]. Further, this literature has discussed that intensification of commercial pressures in the profession of pharmacy are resulting in pharmacists feeling pressure to make unethical decisions that may be in conflict with their moral values [36]. This evidence only serves to strengthen the argument of growing moral injury distress in health care providers. With the growing concern over the moral crisis faced by providers, the authors were interested in how questionable practices by key organizations undergoing public scrutiny would stand through a framework more commonly aligned with the decision-making of providers, that of an ethical analysis.

When considering potential economic and legal solutions, an ethical analysis would provide a novel and grounded framework for understanding the potential ethical considerations by providers as well as broader context for stakeholders. Moreover, there exists a gap in the literature relating to how such organizational practices in health care, and those of PBMs in particular, might fare under ethical examination. Therefore, as a first step towards bridging this gap, this study sought to investigate the ethical nature of PBM practices using ethical decision-making models.

Ethical issues in health care, including pharmacy, are commonly discussed using four core bioethical principles of beneficence, non-maleficence, autonomy and justice [37,38] as well as with professional codes of ethics [34]. In addition, some pharmacy specific texts have included fidelity and truthfulness as newer ethical principles with application and relevance [39]. However, work by Cooper et. al. described that much previous empirical ethical work in pharmacy commonly used these principles solely due to normative consideration rather than because these values are reported by pharmacists themselves. These authors observed pharmacists used a range of ethical values during the reasoning process, and that various ethical approaches may have relevance to pharmacy dilemmas. Moreover, research has found that even when provided a four-stage process guideline, pharmacists displayed limited abilities in ethical reasoning [39]. Cooper and colleagues’ open-ended empirical approach hoped to capture the complexity and nuance in pharmacist decision-making, however, it was found that pharmacists frequently reasoned dilemmas in legal terms and lacked ethical awareness. Therefore, given the overall lack of literature on ethical theory in pharmacy combined with the possibility of criticism on the application of normative ethical theory (i.e., the four major bioethical principles), it is prudent to take a comprehensive approach.

There is some ethical theory literature which proposes the use of multiple frameworks to compare contextual differences from a clinical versus an organizational standpoint [28,33]. Further, this work argues complexities of health care rarely allow one model to capture value differences among all stakeholders. Thus, it would be of interest to use multiple ethical models in order to compare potential value differences encased in various ethical scenarios. The literature suggests that pharmacists may need additional guidance for ethical decision-making in practice [39] and that there have been calls for integration of novel ethical schemas for grappling with complex and especially commercial aspects of health care [34]. As a result, the authors chose to use established decision-making models based upon a diversity of ethical theories and principles.

Philosophical decision-making models assist with debate and identification of action steps given an ethical issue. Work by Hammaker and Knadig, have summarized eight decision-making models based on the major philosophers cited by the U.S. Supreme Court, the U.S. Court of Appeals, and the state Supreme Courts since 2010 [40]. These models comprehensively cover teachings and writings of philosophers that included: Kant, Rawls, Dworkin, Bentham, Mill, Confucius, Socrates/Plato/Aristotle, Bradley, Epictetus, and Sartre [40] (p. 8). These decision-making models were appealing to use as a framework in this study because they have been used extensively in the U.S. legal system, which the literature has shown is important to pharmacists [39]. They include principles from deontology, teleology, and utilitarianism as well as others. Regarding the eight accepted decision-making models, this study chose six to use for analysis. After consideration, two models were excluded from the final analysis due to inapplicability and ambiguity of context when attempting to create a consistent assessment. In place of the two excluded decision-making models, the authors chose to examine how two professional codes of ethics might assess each of the issues. Provider codes of ethics are also referenced in the literature as ways health care providers are taught and therefore utilize ethical values in practice [19,34].

In sum, because there has been a lack of ethically-based work surrounding organizations involved in the high costs of drugs and that a gap remains in ethics-based pharmacy research, this study used scrutinized PBM practices to comprehensively consider these issues within an ethical framework. Therefore, the objective of this study was to systematically apply multiple ethical decision-making models to specific PBM practices and determine their ethical nature.

## 2. Methods

### 2.1. Conceptualization

In this study, several types of controversial PBM practices were chosen for evaluation. PBM practices were chosen based those receiving evaluation in U.S. government issued reports [7,14]. First, this study identified if the practice was receiving scrutiny and a potential cause for increasing drug pricing [14] (pp. 32–38). Second, the authors assessed if the practice could stimulate changes in the drug supply chain [7] (pp. 26–32). And finally, the authors predicted if this practice or related changes in the drug supply chain could present ethical concerns to providers. If a PBM practice was able to sufficiently meet all elements for inclusion, it was evaluated using a systematic approach to determine its ethical nature. This study was limited to a maximum of five ethical scenarios to ensure a thorough analysis. The scenarios considered the market environment, including the purpose of the organization, typical goals (i.e., cost-effectiveness and profit-maximization) and motivations of the involved stakeholders. The scenarios considered in this study were purposely hypothetical and therefore did not use actual cases, or details from any specific organization or company. The models have differences in value considerations, yet all of which are considered a reputable means of determining an ethical decision with health care and the legal systems [40]. This comprehensive approach provides a framework for future work. The chosen ethical scenarios are detailed in Table 1.

### 2.2. Analytical Approach

This study used a systematic ethical analysis of PBM practices utilizing decision-making models [40] (pp. 4–35) based upon a foundation of ethical philosophies. The six decision-making models were applied to all five selected PBM practices in a detailed step-wise assessment. The foundational ethical philosophies that comprise the six models are fully described in chapter one of the Hammaker and Knadig text [40]. These were the utility model (utilitarianism), exceptions model, choices model, justice model, common good model, and virtue model. In the utility model, maximizing good and minimizing harm are the foci of this ethical determination. The exceptions model is a unique framework to consider the ethical nature if an individual exception became the standard or norm for all. The choices model places a focus on how decisions are made, with an emphasis being placed on moral respect for individual choices. The justice model, commonly applied in health care, involves the consideration of the distribution of limited resources. The common good model is similar to the utility model, but rather than focus on benefits to individuals, it asks to focus on how a decision might benefit everyone. Finally, the virtue model focuses on core values aspired to by the decider, and determinations whether a given scenario helps or hinders one towards reaching these values. Examples of core values of health professionals may include characteristics of compassion, honesty, or self-control. Complete descriptions of the specific step-wise framework for application of the models were included in Table S1A–F, in the Supplemental Materials. More specific details about the foundational ethical theory and foundational philosophies for each of the models can be found the Hammaker and Knadig text.

To demonstrate reproducibility, the following outlines an example of how a step-wise application of each ethical model would be conducted. These steps and application responses were applied to the five chosen ethical scenarios. They were determined by the authors separately, and then together through detailed discussion until a consensus for each scenario was reached. In this application, the steps outlined for the justice model (Table S1D, Supplementary Materials) were applied to the PBM practice known as exclusion lists.

The justice model contains five steps: (Step 1) Define the distribution of resources by determining who is getting the benefits and burdens in the situation. Should those who get benefits also share burdens? (Application Response 1) The purpose of the PBM’s formulary cost structure is to fairly distribute prescription benefits and burdens (i.e., costs) across all plan members, assuming reasons for any inequality are fair and just. (Step 2) Once the distribution is known, establish which criterion for distribution would be the fairest and justify why it would be most fair in the situation. (Application Response 2) The fairest distribution is typically through PBM authorized prescription cost structures based on medication tiering and copayment agreements with plan members. The use of these structures and formularies allows for fair distribution based on principles of cost-effectiveness medication use. (Step 3) Select a framework to decide what is fair if disagreement persists over which outcome is fair or over which criterion for inequality is best in the situation, then choose a framework to decide what is fair. (Application Response 3) The framework used to determine fairness has been built into the PBM authorized formulary structure and plan sponsor pricing. Factors such as employment, premiums, maximum out-of-pocket expenses, exclusion lists, etc. all have a role in pricing and payment. Following agreed upon terms of the authorized formulary will most consistently produce a fair outcome. (Step 4) Make an ethical decision. Decide whether an action will produce a fair distribution and why. (Application Response 4) This practice is deemed ethical. Exclusion lists are applied to prevent inconsistencies within authorized cost structures to maximize cost-effectiveness for plan members. (Step 5) Monitor and reassess when applicable.

Ethical scenarios were also assessed using the Pharmacist Code of Ethics and the Physician Code of Ethics [41,42]. These two professional codes of ethics were chosen because of the potential impact of these health care providers as fiduciary advocates in the pharmaceutical market. For the analysis with the professional codes, the authors added the total number of sections (i.e., Section I, Section II.) finding the practice as ethical, to the total number finding the practice as unethical, to calculate the overall ethical determination. In other words, the authors assessed whether each section would call the practice ethical or unethical. Inclusion of other health provider’s codes of ethics was beyond the scope of the study’s objective. There was no weighting assigned to these assessments, each model or code section stood on its own merits. Once an ethical nature was determined using each model and an individual code of ethics, the resulting ethical determinations were totaled.

## 3. Results

The PBM practice that was most likely to be found ethical by the models included point of sale rebates. Exclusion lists were equally found to be both ethical and unethical, depending on the model applied. In contrast, a clear majority of ethical models found market consolidation and gag clauses to be unethical. Additionally, all ethical models unanimously found that the fluctuation of pharmacy reimbursement rates to be unethical.

The ethical models found different results for each PBM practice, with the exception of the common good and choices models, which made identical ethical assessments. Further, the codes of ethics applied also found differing assessments. The Physician Code of Ethics aligned most closely with the utility and the justice models. In contrast, the Pharmacist Code of Ethics aligned most closely with the choices and common good models.

A complete summary and cross-tabulation of ethical assessments for the models and codes of ethics can be found in Table 2, below.

## 4. Discussion

This study systematically applied multiple ethical decision-making models to a variety of PBM practices. The results showed a novel assessment of ethically questionable PBM practices with mixed findings on their ethical nature. The PBM practices were determined to be simultaneously ethical and unethical depending on the varied considerations for each applied model. The application of the codes of ethics of pharmacists or physicians further created comparisons for these results.

In consideration of the PBM practices, exclusion lists produced the most varied findings when the ethical models were applied. More consistent results were established with all other PBM practices, which were predominately deemed unethical. Point-of-sale rebates were the only PBM practice with a consistent ethical determination. This practice has been more recently scrutinized and more information will develop with changes in the market that could change results [43]. Future assessment is warranted for this ethically questionable scenario.

The majority of PBM practices resulted in an unethical determination. These results can be attributed, in part, to the ethical philosophies in which the decision-making models are based. Generally, philosophical renderings consist strictly of a two-sided pendulum, one beneficence (i.e., ensuring welfare) and the other maleficence (i.e., opposes welfare). When applying the theories or models, a decision must be made with little acceptance or consideration of an ethical spectrum. However, the context does matter and could change the decision. It has also been noted that vagueness of ethical codes is also a cause of conflict for a consensus of interpretation [19] (pp. 4–35). Many PBM actions were simultaneously ethical and unethical dependent on the applied philosophical model. However, unethical results are more prevalent because greater significance tends to be placed on acts of beneficence or maleficence with a direct effect on individuals rather than societies. Whereas, the objective of PBM organizations is the cost-effective management of medication use for an entire covered group of people, or a population, and therefore more closely align with the needs of a broader society than an individual.

More specifically, the common good model and the choices model produced identical ethical determinations for all PBM practices under ethical assessment. The models, although different in structure and application, produced identical results due to a shared emphasis on the individual within a society. The common good model determines ethical nature through the distribution of benefits and burdens of individuals within a society, as well as the sustainability of the distribution. The choices model requires individuals to be given an individualized determination of their life outcome via specific choices and values. The choices model and the common good model both ethically assess society through an individualized perception. The two models strongly favor an unethical determination of recently scrutinized PBM practices.

The utility model and common good model produced the most contradicting results among the ethical models. Regarding the five practices assessed, three showed disagreement. The utility model and the common good model are nearly identical in their structure and application, however found different results. The models assess foreseeable benefits and burdens resulting from an action. Then, the models determine the most beneficial option available as an ethical decision. However, the important distinction is the utility model only considers those immediately affected by an action, whereas the common good model considers the benefit and burden to all of society and also accounts for unintentional and non-immediate benefits and burdens. The subtle difference is significant when determining results. An example of such disagreement is also demonstrated when assessing gag clauses. The utility model determines gag clauses to be justified and therefore ethical because they produce the best overall outcome and the greatest long-term benefit to those affected. The common good model determines gag clauses to conflict with the pharmacist’s ethical obligation, which prioritizes the importance of maintaining a beneficial and truthful relationship with patients. The ideologies upheld by a pharmacist’s ability to communicate transparently to patients contribute more common good to society than any common good deriving from the PBM’s gag clause. In this scenario, the common good is the general condition that all patients have equal access to a quality relationship with their pharmacist.

The differences among the models for the ethical scenario of market consolidation further demonstrate how subtle philosophical orientation can provide context to an ethical determination. Both the utility model and virtue model supported market consolidation but for different reasons. For example, the utility model supported market consolidation as ethical because of the potential effect on the distribution of benefits and burdens of those immediately affected by the action. Those immediately affected are likely to already hold a prescription benefit plan within the established system. The consolidation of entities within the pre-established market will further benefit plan holders due to increased access to affordable care through plan participation. Those who do not hold plans within the system will not have any direct benefit and are likely burdened by a decreased supply within the market upon entry. However, the utility model does not take those individuals into consideration when making an ethical determination due to a lack of immediate effect. The virtue model focuses primarily on the aspirations of a professional and secondarily on the action itself. More specifically, the virtue model emphasizes a health care professional’s ability to deliver high-quality and equitable care to the patient population. This emphasis on delivery of care supports the consolidation of the market. Consolidation allows for near universal availability, coverage, and access to prescription benefits, which benefits both the health care professionals and patients financially and clinically. However, the patient must have sufficient resources to gain access to the market in order to experience the added benefits consolidation offers. The common good and choices models place emphasis on individual assessment. In general, consolidation usually places importance on the benefit to a population or society rather than an individual perception.

The Physician’s Code of Ethics, virtue model, justice model, and exceptions model produced mixed results. In contrast, the utility model, choices model, common good model and Pharmacist’s Code of Ethics were more consistent. An interesting finding was a lack of alignment between the Pharmacist’s Code of Ethics and the Physician’s Code of Ethics. This could be due to specificities within each code of ethics. The physician’s code appears to have a broadened perspective, emphasizing the value of societal health as well as the individual patient. For example, Sections VII and IX (Table S2B, Supplementary Materials) are both pointing towards the broader community and societal issues of health care access to all and to public health. Whereas, the pharmacist’s code has an emphasis focused more specifically on individual patients. For example, the only mention of broader society is in Section VIII (Table S2A) and this section speaks of a balance towards the broader societal distribution of resources with individual patients. The similar but differing ethical values could be viewed as a system of checks and balances between types of care providers or could be viewed as a way to better understand the foundational values of each unique profession. It would be of further interest to compare ethical codes for additional health professions.

The future success of quality care relies on providers who are willing to actively assess the changing landscape of the health care system and confront potentially unethical practices. Grass-roots advocacy by health providers have had, and continue to have, the potential to make changes in health care both from a regulatory and organizational standpoint. Therefore, it is critical for providers to be knowledgeable about current practices impacting foundational values that guide their work. Despite the multiplicity of theory-based ethical models as well as ethical codes among the health professions, it is important that providers are supported when complex problems in health care infringe on their moral values. Further, ethical models and professional codes can assist in guidance, but should be used cautiously if applied singularly or in a simplified dichotomous (i.e., ethical/unethical) or authoritative way. Therefore, this study provides an example of how to use theory-based models and multiple professional codes to deepen the understanding of current ethical problems in practice. Finally, in pharmacy as in other health professions, ethical standards can have important legal implications and thus, make practical application essential.

## 5. Limitations

The limitations of this study can be categorized into a few areas. Despite a structured step-wise process, there remains subjectivity in decision-making application and the rationales for determinations. Others following a similar assessment may or may not have greater insight and exposure to the actions and therefore may conclude alternative findings compared to those found in this study. Second, there is an inherent lack of certainty in organizational assumptions based on cost-effectiveness information and actuarial analysis as to whether or not a specific practice brings greater benefits or burdens to a population. The authors propose the assessment in this study to be value-neutral but remains hypothetical, nonetheless. For example, many of these determinations were made under the assumption that both PBMs and plan sponsor organizations value efforts to lower the cost of health care delivery in the U.S. while maintaining profit expectations of both employees and investors. These assumed values are an area of ambiguity, differ between organizations, and may undergo fluctuation based on environmental factors in the marketplace. For example, exclusion lists were initially expected to decrease pharmaceutical expenditures by limiting market availability to drug manufactures. However, further market development revealed exclusion lists do not always produce decreased expenditures [15].

Additionally, fundamental assumptions were made of both PBMs and the closely linked organization of plan sponsors. A fundamental concept of PBM organizations is to increase purchasing power when negotiating with manufacturers to provide discounted drug pricing. This discount is passed to the plan sponsor via rebates. PBMs can, therefore, produce savings on behalf of the plan sponsor from these negotiated rebates. In theory, the plan sponsor uses the rebates to reduce the costs to all of the plan members. However, some PBMs have openly claimed all rebates are retained, with no distribution to plan members in any way [44] (p. 12). Rebates are assumed to be retrospectively applied to future pricing. However, it is possible that these rebates are not accounted for in pricing models. The potential for organizational variations must be considered when assessing the ethical nature of organizational practices [23]. Recent policy proposals in the U.S.’s Centers for Medicare and Medicaid Services (CMS) have furthered market uncertainty. Recent analyses have been unable to accurately predict potential financial outcomes of developed regulations due to irregular responses from manufacturers, plan sponsors, and PBMs [45]. However, supply chain transparency and point-of-sale rebates are considered market trends that could benefit the overall population.

## 6. Conclusions

This analysis fills a gap in the literature to use ethically-based frameworks to assess organizations involved in the high costs of pharmaceuticals. To the knowledge of the authors, an ethical perspective has not been previously applied to scrutinized organizational PBM practices. Theory-based ethical decision-making models provided a variety of perspectives that offer practical context to current dilemmas in health care. Depending on the applied model, the assessed scenarios resulted in a mix of ethical and unethical determinations. Despite variation across applied models, some practices were predominately ethical or unethical. Point of sale rebates were consistently determined as ethical, whereas market consolidation, gag clauses, and fluctuation of pharmacy reimbursements were all predominantly determined as unethical. The application using provider codes of ethics created additional and diverse comparison for the analysis. The codes of ethics were different in their overall assessment for ethical determination with the physician’s code aligning more closely with values of societal benefit, whereas the pharmacist’s code aligned more closely with patient-centric values.

This approach can be applied across many areas of health care as a mechanism allowing for the productive analysis and open-minded deliberations of health care issues. The systematic assessment of ethical dilemmas help guide providers towards grounds for concrete argumentation when applied with a fundamental understanding of involved stakeholders and realistic solutions. It remains imperative that health care professionals retain accountability towards addressing difficult ethical questions in practice. Moreover, given the potential moral distress encountered as professionals closely tied with both the clinical and fiduciary aspects of medication use, it is essential that pharmacists remain committed to ethical reasoning amidst a rapidly evolving pharmaceutical market.

## Figures and Tables

**Table 1:** Ethical Scenarios.

**Gag Clauses**	A commercial contract between a pharmacy benefit manager and a network of pharmacies prohibiting a pharmacist from informing a patient of alternative prescription purchasing options [16]
**Fluctuation of Pharmacy Reimbursement Rate**	PBM determined redirect and indirect remuneration (DIR) fees assessed to pharmacies retrospectively dependent upon incentive-based performance [17] (p. 33)
**Exclusion Lists**	List of branded prescription medications to be excluded from insurance coverage [15]
**Market Consolidation**	Large mergers and acquisitions have been increasing between pharmacy benefit management organizations and other organizations within health care [7] (pp. 30–32)
**Point-of-Sale Rebates**	A commercial contract between pharmacy benefit manager and discount card providers offering cash-paying patients discounted prescription pricing [18]

**Table 2:** Ethical Analysis.

Scenario & Model	Market Consolidation	Pharmacy Reimbursement Rate	Gag Clauses	Exclusion Lists	Point of Sale Rebates	Totals
**Utility**	Ethical	Unethical	Ethical	Ethical	Ethical	Ethical: 4 Unethical: 1
**Exceptions**	Unethical	Unethical	Ethical	Ethical	Unethical	Ethical: 2 Unethical: 3
**Choices**	Unethical	Unethical	Unethical	Unethical	Ethical	Ethical: 1 Unethical: 4
**Justice**	Unethical	Unethical	Unethical	Ethical	Ethical	Ethical: 2 Unethical: 3
**Common Good**	Unethical	Unethical	Unethical	Unethical	Ethical	Ethical: 1 Unethical: 4
**Virtue**	Ethical	Unethical	Unethical	Unethical	Ethical	Ethical: 2 Unethical: 3
**Pharmacist**	Unethical	Unethical	Unethical	Unethical	Ethical	Ethical: 1 Unethical: 4
**Physician**	Unethical	Unethical	N/A	Ethical	Ethical	Ethical: 2 Unethical: 2
**Totals**	Ethical: 2 Unethical: 6	Ethical: 0 Unethical: 8	Ethical: 2 Unethical: 5	Ethical: 4 Unethical: 4	Ethical: 7 Unethical: 1	Ethical: 15/39 Unethical: 24/39

## Supplementary Materials

The following are available online at https://www.mdpi.com/2226-4787/7/2/65/s1, Table S1A: Utility Model, Table S1B: Exceptions Model, Table S1C: Choices Model, Table S1D: Justice Model, Table S1E: Common Good Model, Table S1F: Virtue Model, Table S2A: Pharmacist Code of Ethics (American Pharmacists Association), Table S2B: Physician Code of Ethics (American Medical Association).
