# Supplementary material for: An Ethical Analysis of Pharmacy Benefit Manager (PBM) Practices
Source: Pharmacy (Basel). 2019 Jun 14;7(2):65. doi: 10.3390/pharmacy7020065 (PMC6631892; doi:10.3390/pharmacy7020065)
Supplement: Supplementary file 1 [file pharmacy-07-00065-s001.pdf]

Supplementary Materials

Table S1A: Utility Model [40, pg 11-13]

Step	Application of the Utility Model
1	Identify the alternative actions that are possible and determine who would be affected by any decision.
2	For each alternative, determine the costs and the benefits for each of the groups affected. (This prediction of short and long term outcomes considers the relative value or marginal utility of the outcomes for different groups of people).
3	Select the action in the current situation that produces the greatest benefits over burdens for everyone affected. If burdens outweigh benefits, the action with the least burden relative to benefit is the best alternative.
4	Predict the consequences of the action for all similar situations. Often what is done in one situation becomes policy for future action. The best alternative maximizes benefits for this and all future situations.
5	Make an ethical decision. If the same action is selected in both steps three and four, then this is the most ethical action. If different actions are selected, decide which individual action will produce the greatest long-term benefits and cause the least burden to everyone affected; this is the most ethical action.
6	Monitor results of the decision and repeat the process as changes occur.

Table S1B: Exceptions Model [40, pg 17-19]

Step	Application of the Exceptions Model
1	Describe the general and specific ethical issues of the situation.
2	Determine what would happen if the exception was adopted by others in similar situations.
3	Decide which exceptions are unacceptable if they become the rule for everyone.
4	Consider what will have to be sacrificed if the exception becomes common. Determine if this is the result that should be created. If not, the exception becomes uncommon, and the exception is not ethical because it is unacceptable for others.
5	Make an ethical decision. If the exception is not practical because everyone would be doing it, then the exception is unethical. Also, if the common adoption of the exception would create an unacceptable result, then the exception is unethical. The reverse is also true. The exception is ethical if not everyone would be requesting exceptions or if common adoption of the exception is acceptable.
6	Monitor the results and repeat the process as changes occur.

Table S1C: Choices Model [40, pg 20-23]

Step	Application of the Choices Model

**1**	Give everyone the freedom to choose what they value. People are not free to make their own choices if they are being forced to choose something they do not value.
**2**	Give everyone the information necessary to know what they value in the situation being considered.
**3**	Make an ethical decision. Decide if the action or situation gives everyone the freedom and the information to choose what they value.
**4**	Monitor the results and repeat the process as changes occur.

Table S1D: Justice Model [40, pg 23-26]

Step	Application of the Justice Model
1	Define the distribution of resources by determining who is getting the benefits and burdens in the situation. Should those who get benefits also share burdens?
2	Once the distribution is known, establish which criterion for distribution would be the fairest and justify why it would be most fair in the situation.
3	Select a framework to decide what is fair if disagreement persists over which outcome is fair or over which criterion for inequality is best in the situation, then choose a framework to decide what is fair.
4	Make an ethical decision. Decide whether an action will produce a fair distribution and why.
5	Monitor the results of the decision and repeat the process all over again as changes occur.

Table S1E: Common Good Model [40, pg 26-30]

Step	Application of the Exceptions Model
1	Identify what specific aspects of the common good are involved by zooming in and then zooming out.
2	Define which specific parts of the common good that depend on the current situation for their functioning could move forward or backward by a change. Some actions will strengthen the common good, some will weaken.
3	Explain the ethical obligation to promote and protect particular aspects of the common good.
4	Determine if the proposed action or situation conflicts with this ethical obligation. Laws and regulations must strengthen the common benefit and protection aspects of them from risks.
5	Make an ethical decision. Determine if the action or situation conflicts with the ethical obligation to contribute to the common good.
6	Monitor the results and repeat the process as change occurs.

Table S1F: Virtue Model [40, pg 30-31]

Step	Application of the Virtue Model
1	Determine whether the situation helps you to or hinders you from becoming the type of professional you most want to be.
2	Establish if the situation corresponds to the industry's reputation or vision of what it would like to be. This image is explained in the health care provider's mission and vision statements, the core values, and the ethics code.
3	Ascertain if the situation improves the delivery of high-quality, equitable health care.

**4**	Make an ethical decision. Actions that correspond to the virtues most people in the health care industry want to be associated with are ethical.
**5**	Monitor the results of the decision and repeat the process as changes occur.

Table S2A: Pharmacist Code of Ethics (American Pharmacists Association) [41]

Section	Application
**Preamble**	Pharmacists are health professionals who assist individuals in making the best use of medications. This Code, prepared and supported by pharmacists, is intended to state publicly the principles that form the fundamental basis of the roles and responsibilities of pharmacists. These principles, based on moral obligations and virtues, are established to guide pharmacists in relationships with patients, health professionals, and society.
**I.**	A pharmacist respects the covenantal relationship between the patient and pharmacist. Considering the patient-pharmacist relationship as a covenant means that a pharmacist has moral obligations in response to the gift of trust received from society. In return for this gift, a pharmacist promises to help individuals achieve optimum benefit from their medications, to be committed to their welfare, and to maintain their trust.
**II.**	A pharmacist promotes the good of every patient in a caring, compassionate, and confidential manner. A pharmacist places concern for the well-being of the patient at the center of professional practice. In doing so, a pharmacist considers needs stated by the patient as well as those defined by health science. A pharmacist is dedicated to protecting the dignity of the patient. With a caring attitude and a compassionate spirit, a pharmacist focuses on serving the patient in a private and confidential manner.
**III.**	A pharmacist respects the autonomy and dignity of each patient. A pharmacist promotes the right of self-determination and recognizes individual self-worth by encouraging patients to participate in decisions about their health. A pharmacist communicates with patients in terms that are understandable. In all cases, a pharmacist respects personal and cultural differences among patients.
**IV.**	A pharmacist acts with honesty and integrity in professional relationships. A pharmacist has a duty, to tell the truth, and to act with conviction of conscience. A pharmacist avoids discriminatory practices, behavior or work conditions that impair professional judgment, and actions that compromise dedication to the best interests of patients.
**V.**	A pharmacist maintains professional competence. A pharmacist has a duty to maintain knowledge and abilities as new medications, devices, and technologies become available and as health information advances.
**VI.**	A pharmacist respects the values and abilities of colleagues and other health professionals. When appropriate, a pharmacist asks for the consultation of colleagues or other health professionals or refers

	the patient. A pharmacist acknowledges that colleagues and other health professionals may differ in the beliefs and values they apply to the care of the patient.
**VII.**	A pharmacist serves the individual, community, and societal needs. The primary obligation of a pharmacist is to individual patients. However, the obligations of a pharmacist may at times extend beyond the individual to the community and society. In these situations, the pharmacist recognizes the responsibilities that accompany these obligations and acts accordingly.
**VIII.**	A pharmacist seeks justice in the distribution of health resources. When health resources are allocated, a pharmacist is fair and equitable, balancing the needs of patients and society.

Table S2B: Physician Code of Ethics (American Medical Association) [42]

Section	Application
**Preamble**	The medical profession has long subscribed to a body of ethical statements developed primarily for the benefit of the patient. As a member of this profession, a physician must recognize a responsibility to patients first and foremost, as well as to society, to other health professionals, and to self. The following Principles adopted by the American Medical Association are not laws, but standards of conduct that define the essentials of honorable behavior for the physician.
**I.**	A physician shall be dedicated to providing competent medical care, with compassion and respect for human dignity and rights.
**II.**	A physician shall uphold the standards of professionalism, be honest in all professional interactions, and strive to report physicians deficient in character or competence, or engaging in fraud or deception, to appropriate entities.
**III.**	A physician shall respect the law and also recognize a responsibility to seek changes in those requirements which are contrary to the best interests of the patient.
**IV.**	A physician shall respect the rights of patients, colleagues, and other health professionals, and shall safeguard patient confidences and privacy within the constraints of the law.
**V.**	A physician shall continue to study, apply, and advance scientific knowledge, maintain a commitment to medical education, make relevant information available to patients, colleagues, and the public, obtain consultation, and use the talents of other health professionals when indicated.
**VI.**	A physician shall, in the provision of appropriate patient care, except in emergencies, be free to choose whom to serve, with whom to associate, and the environment in which to provide medical care.

**VII.**	A physician shall recognize a responsibility to participate in activities contributing to the improvement of the community and the betterment of public health.
**VIII.**	A physician shall, while caring for a patient, regard responsibility to the patient as paramount.
**IX.**	A physician shall support access to medical care for all people.
